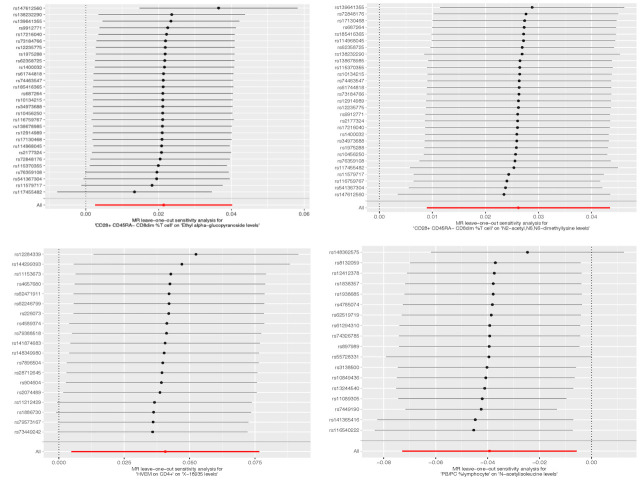# Genetically predicted N-acetylisoleucine levels mediate the association between PB/PC% lymphocyte and normal pressure hydrocephalus: A mediation Mendelian randomization study

**DOI:** 10.1097/MD.0000000000049395

**Published:** 2026-06-19

**Authors:** Chang Liu, Junqiang Wang, Yexin Yuan, Peng Long, Gelei Xiao, Cheng Wang

**Affiliations:** a Department of Encephalopathy (Neuromedicine), Ningxiang Hospital of Traditional Chinese Medicine, Changsha, Hunan, PR China; b Department of Neurosurgery, Xiangya Hospital, Central South University, Changsha, Hunan, PR China; c Diagnosis and Treatment Center for Hydrocephalus, Xiangya Hospital, Central South University, Changsha, Hunan, PR China; d National Clinical Research Center for Geriatric Disorders, Xiangya Hospital, Central South University, Changsha, Hunan, PR China; e Department of Neurosurgery, Changsha Hospital of Traditional Chinese Medicine (Changsha Eighth Hospital), Changsha, Hunan, PR China.

**Keywords:** immune cell phenotypes, mediator, Mendelian randomization, normal pressure hydrocephalus, plasma metabolites

## Abstract

Normal pressure hydrocephalus (NPH) is defined by a clinical triad of gait disturbances, urinary incontinence, and cognitive decline. The mechanisms underlying NPH are complex, and emerging evidence suggests that immune cells significantly contribute to the neuroinflammatory processes associated with this condition. However, research elucidating the causal relationships and mechanisms between them remains limited. A mediation Mendelian randomization (MR) was employed to investigate the causal relationship among immune cell phenotypes, NPH, and potential mediating plasma metabolite levels. Using publicly available data from genome-wide association studies, we explored 731 immune cell phenotypes, 1400 plasma metabolite levels, and NPH through a 2-sample MR approach. Furthermore, we employed a mediation MR approach to evaluate the mediating effect of plasma metabolite levels on the relationship between immune cell phenotypes and NPH. Our analysis identified 6 immune cell phenotypes correlated with NPH, with 3 acting as protective factors and 3 as risk factors, none of which showed an inverse causal relationship with NPH. Additionally, we established a causal association between 17 plasma metabolite levels and NPH. Among the 12 identified metabolites, 6 were classified as protective factors and 6 as risk factors. Mediation MR analysis revealed a mediating effect of N-acetylisoleucine levels on the association between plasmablasts/plasma cells% lymphocytes and NPH. Our findings confirm causal relationships linking 6 immune cell phenotypes and 17 plasma metabolite levels to NPH and the protective role of plasmablasts/plasma cells% lymphocytes in NPH through reducing N-acetylisoleucine levels, thereby providing new insights into the pathophysiological mechanisms underlying NPH.

## 1. Introduction

Normal pressure hydrocephalus (NPH) is defined by a clinical triad of gait disturbances, urinary incontinence, and cognitive decline.^[1]^ It is characterized by a normal opening pressure during lumbar puncture and disproportionate ventricular enlargement in relation to cortical atrophy.^[2]^ Presently, treatment strategies for NPH mainly involve surgical interventions and medication. Nonetheless, due to the complex nature of its etiology, these approaches may have limited efficacy in certain patients, highlighting the need for deeper investigations into the pathophysiology of NPH.^[3]^

Research has shown that immune cells play key roles in various neurodegenerative disorders, and that inflammatory responses potentially contribute to the development of NPH.^[3,4]^ For instance, microglia, the primary immune cells of the central nervous system (CNS), are essential for maintaining tissue homeostasis and supporting brain development.^[5,6]^ Excessive or persistent activation has been observed in several neurodegenerative conditions, including Alzheimer disease, Parkinson disease, Huntington disease, and amyotrophic lateral sclerosis.^[7]^ In addition to microglia, B cells also contribute to CNS immunity. They enhance immune responses and regulate excessive immune activity through secreting antiinflammatory factors. Nevertheless, the interactions between NPH and immune cells are still poorly understood, highlighting the need for additional research to clarify these mechanisms. Metabolic dysregulation can initiate a series of changes in brain injury.^[8–10]^ Changes in glucose metabolism, serine, 2-hydroxybutyrate, glyceraldehyde, and N-acetylneuraminic acid have been observed in the CSF and serum of NPH patients, helping to better understand the disease’s onset and progression.^[11,12]^ Similar metabolic abnormalities have been observed in other neurological conditions. However, the relationship between altered plasma metabolite levels and the neurological deficits associated with NPH remains largely unclear and warrants further investigation. Existing studies have shown that metabolites can mediate the effects of immune cells on disease.^[13]^ Understanding these associations and the relationships among immune cells, metabolites and NPH could aid in the prevention and management of NPH.

In Mendelian randomization (MR), the random allocation of genetic variants closely resembles the natural randomization in randomized controlled trials. Compared with traditional observational studies, MR analyses are less affected by confounding factors and reverse causation.^[14]^ As a result, MR provides an important methodological framework for exploring causality when randomized clinical trials are not feasible.

While multiple hypotheses have been proposed regarding the pathogenesis of NPH,^[15,16]^ research on its causal mechanisms remains limited. Therefore, in this study, we performed a 2-sample MR analysis to evaluate the causal association between immune cell phenotypes and NPH. We then explored the correlation between plasma metabolite levels and NPH. Finally, we used mediation MR to assess whether plasma metabolites mediate the effect of immune cell phenotypes on NPH. We clarified how immune cell phenotypes and plasma metabolites influence the development of NPH and identified potential therapeutic targets.

## 2. Materials and methods

### 2.1. Study design

**Figure 1.**
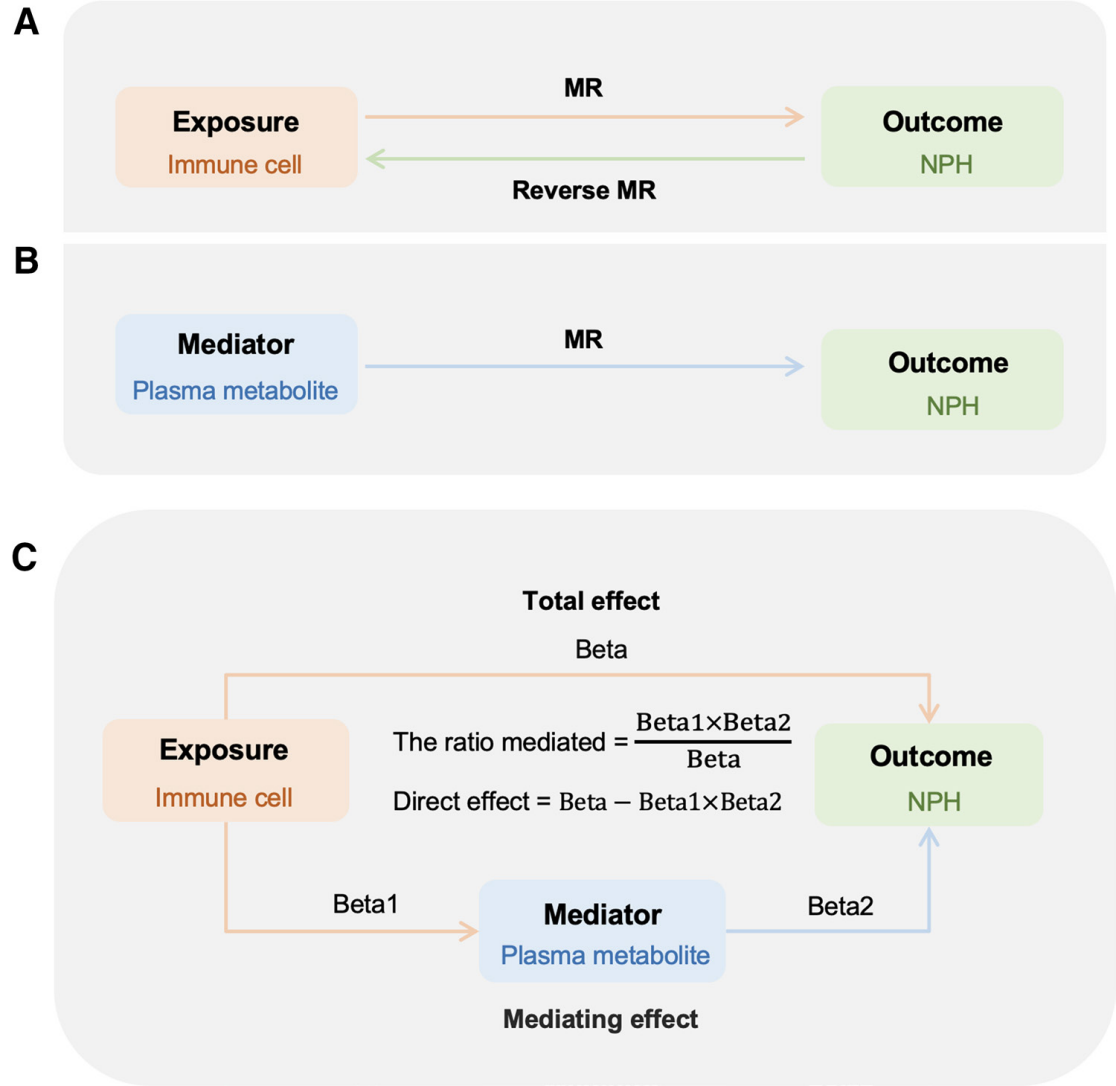
A flowchart of MR and mediation MR analysis. (A) Immune cell phenotype and NPH for MR and reverse MR analysis. (B) Plasma metabolite levels as an exposure and NPH as an outcome for MR. (C) A mediation MR analysis evaluating the role of 1400 plasma metabolite levels as mediators in the relationship between 731 immune cell phenotypes and NPH. MR = Mendelian randomization, NPH = normal pressure hydrocephalus.

In this study, we first used 2-sample MR to explore the potential causal relationship between 731 immune cell phenotypes and NPH. Subsequently, we performed reverse MR to evaluate whether NPH might causally influence immune cell phenotypes and to determine whether mediation MR would be appropriate. Finally, we conducted mediation MR to assess whether any of the 1400 plasma metabolites acted as mediators between immune cell phenotypes and NPH (Fig. 1). Throughout all analyses, single nucleotide polymorphisms (SNPs) were utilized as instrumental variables (IVs). We selected these IVs based on 3 core assumptions: (1) the IVs are directly associated with the exposure of interest; (2) they are independent of any confounding factors between the exposure and the outcome; (3) they affect the outcome only through the exposure.^[17]^ All studies included in our analysis had received approval from the relevant institutional review boards, and informed consent was obtained from all participants.^[18,19]^

### 2.2. Data sources

The genome-wide association study (GWAS) data for NPH were obtained from the FinnGen study (https://r11.finngen.fi). FinnGen is a large genomic project that links genetic variants with health data to enhance the understanding of disease mechanisms and susceptibility.^[20]^ According to FinnGen, NPH cases are identified based on the International Classification of Diseases, 10th edition. Individuals with a hospitalization record coded as G91.2 are classified as NPH cases. In the 11th round of FinnGen, the dataset included 1553 NPH cases and 451,091 controls, all of European ancestry.

Summary statistics for 731 immune cell phenotypes were readily available in the GWAS database (https://gwas.mrcieu.ac.uk/), with identifiers ranging from ebi-a-GCST90001391 to ebi-a-GCST90002121. These data were derived from a cohort of 3757 individuals of European descent. The dataset includes 118 absolute cell counts (AC), 389 median fluorescence intensities representing surface antigen levels, 32 morphological parameters, and 192 relative cell counts.^[19]^ These immune cell phenotypes cover 7 panels, including B-cell, dendritic cell (DC), T/B/NK cell , Treg, myeloid cell, maturation stages of T cell, and monocyte.

GWAS data for 1400 plasma metabolite levels were obtained from the 2023 Canada Longitudinal Study on Aging (CLSA). This cohort included 8299 participants and measured 1091 plasma metabolites and 309 metabolite ratios.^[18]^ The study investigated the genetic basis of metabolites and their relevance to common diseases, offering potential implications for therapeutic development. The dataset is available in the GWAS Catalog (https://www.ebi.ac.uk/gwas), with identifiers from GCST90199621 to GCST90201020, all based on the European population.

The original GWAS adjusted association analyses for age, sex, and genetic principal components to control for population stratification, and also accounted for relevant technical covariates in trait measurement.^[18,19]^

### 2.3. Selection of IVs

Following the 3 core assumptions described earlier, we selected IVs from the publicly available GWAS datasets. Based on recent research,^[21–23]^ we established a significance threshold of 1 × 10^−5^ for immune cell phenotypes and plasma metabolites to identify SNPs. Concurrently, to ensure the independence of SNPs, we excluded those exhibiting linkage disequilibrium (correlation coefficient *r*^2^ < 0.001) within a genetic distance of 10,000 kb.^[23]^ For the reverse MR analysis, we applied a threshold of 5 × 10^−6^ to select SNPs that are closely associated with NPH. The same linkage disequilibrium criteria (10,000 kb, *r*^2^ < 0.001) were used to further minimize correlation among variants. For each SNP that met the specified criteria, we calculated the *R*^2^ and *F*-statistic. SNPs with an *F*-statistic < 10 were considered weak IVs and were excluded from the analysis.^[24]^

### 2.4. Statistical analysis

We analyzed the data using the TwoSampleMR package (version 0.6.6) in RStudio software (version 4.2.2). We employed 5 MR analysis methods: inverse variance weighted (IVW), MR-Egger, weighted median, simple mode, and weighted mode. Among these, IVW served as the primary approach for assessing causality. To ensure the robustness of our findings, we performed pleiotropy, heterogeneity, and leave-one-out analysis. We evaluated instrumental variable pleiotropy with the MR-Egger intercept. For heterogeneity, we used the Cochran *Q* test and interpreted *P*-values >.05 as showing no evidence of either pleiotropy or heterogeneity. To check whether any single SNP drove the overall findings, we performed a leave-one-out analysis by removing each SNP 1 at a time. In the mediation analysis, we initially calculated the total effect of immune cell phenotype on NPH (Beta). We then estimated the effect of the immune cell phenotype on plasma metabolite levels (Beta 1) and the effect of plasma metabolite levels on NPH (Beta2). We defined the mediating effect as Beta 1 × Beta 2. The direct effect was derived by subtracting the mediating effect from the total effect. The mediation ratio was calculated by dividing the mediating effect by the total effect (Fig. 1). A mediating effect existed when Beta 1 × Beta 2 and Beta showed associations in the same direction.^[25]^ To reduce false-positive results arising from multiple testing, we applied false discovery rate correction to all *P*-values.

## 3. Results

### 3.1. Genetic causality of immune cell phenotypes on NPH

**Figure 2.**
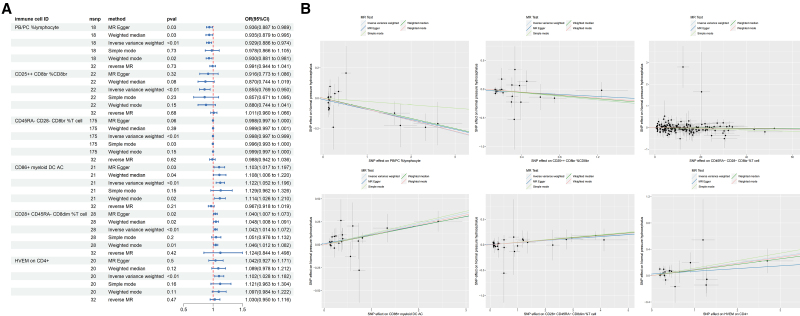
The association between immune cell phenotypes and NPH using 5 MR methods and the results of reverse MR between NPH and immune cell phenotypes. (A) Forest plot of causality between 6 immune cell phenotypes and NPH. (B) Scatter plots of 6 immune cells. MR = Mendelian randomization, NPH = normal pressure hydrocephalus.

In our study, we screened instrumental variables and identified 18,728 SNPs that met the significance threshold for the 731 immune cell phenotypes. The lowest *F*-statistic among these variants was 19.537, indicating that all selected SNPs were strong instruments. Using the IVW method as our primary approach for MR analysis, we performed a 2-sample MR analysis that uncovered 6 immune cell phenotypes associated with NPH. We also conducted tests for heterogeneity and pleiotropy, as summarized in Table 1. Our findings indicated that certain immune cell phenotypes, such as CD86 + myeloid DC AC (OR = 1.122, 95% CI = 1.052–1.196, *P*_adj = .003), CD28 + CD45RA-CD8dim% T cell (OR = 1.042, 95% CI = 1.014–1.072, *P*_adj = .006), HVEM on CD4 + (OR = 1.102, 95% CI = 1.028–1.182, *P*_adj = .006) increased the risk of hydrocephalus, however, plasmablasts/plasma cells (PB/PC)% lymphocyte (OR = 0.929, 95% CI = 0.886–0.974, *P*_adj = .006), CD25++CD8br% CD8br (OR = 0.855, 95% CI = 0.769–0.950, *P*_adj = .006), CD45RA-CD28-CD8br% T cell (OR = 0.998, 95% CI = 0.997–0.999, *P*_adj = .006) were potential protective factors. Results from the other 4 MR methods were consistent with the IVW findings for all 6 immune cell phenotypes, and no evidence of heterogeneity or pleiotropy was observed for any of the phenotypes (Fig. 2). Finally, the leave-one-out analysis showed that no outlier SNPs influenced the overall results (see Figure S1, Supplemental Digital Content 1, which demonstrates the result of the leave-one-out analysis).

**Table 1:** MR analysis of the causal relationship between immune cell phenotypes and NPH.

Exposure	Method	*N*snp	Beta	SE	*P*-val	*P*-val_adj	Pleiotropy	Heterogeneity
PB/PC% lymphocyte	IVW	18	−0.074	0.024	.002	.006	.57	.61
CD86 + myeloid DC AC	IVW	21	0.115	0.033	<.001	.003	.53	.98
CD28 + CD45RA-CD8dim% T cell	IVW	28	0.042	0.014	.004	.006	.75	.98
CD25++CD8br% CD8br	IVW	22	−0.157	0.054	.004	.006	.32	.65
CD45RA-CD28-CD8br% T cell	IVW	175	−0.002	0.001	.005	.006	.82	.81
HVEM on CD4+	IVW	20	0.097	0.036	.006	.006	.26	.76

IVW = inverse variance weighted, MR = Mendelian randomization, NPH = normal pressure hydrocephalus.

### 3.2. Genetic causality of NPH on immune cell phenotypes

In our study, we conducted a reverse MR analysis, using NPH as the exposure factor and 5 immune cell phenotypes as the outcome factors. Notably, OR for the CD45RA–CD28–CD8br% T cell was too close to 1, leading us to exclude it from further analysis. Our results showed that NPH did not have any inverse causal relationship with 5 immune cell phenotypes (*r*-*P* > .05) (Fig. 2). In other words, NPH did not meaningfully alter these immune cell traits, which allowed us to move forward with the mediation MR analysis. Additionally, our tests for heterogeneity and pleiotropy showed no significant results (*P* > .05), confirming the absence of notable heterogeneity and pleiotropy in our data (Table 2).

**Table 2:** Reverse MR analysis of NPH and immune cell phenotypes.

Outcome	Method	*N*snp	Beta	SE	*P*-val	*P*-val_adj	Pleiotropy	Heterogeneity
PB/PC% lymphocyte	IVW	32	−0.009	0.025	.73	.73	.69	.90
CD86 + myeloid DC AC	IVW	32	0.011	0.027	.68	.73	.56	.60
CD28 + CD45RA-CD8dim% T cell	IVW	32	−0.012	0.024	.62	.73	.63	.72
CD25++CD8br% CD8br	IVW	32	−0.033	0.027	.21	.73	.23	.85
HVEM on CD4+	IVW	32	0.030	0.041	.47	.73	.40	.82

IVW = inverse variance weighted, MR = Mendelian randomization, NPH = normal pressure hydrocephalus.

### 3.3. Genetic causality of plasma metabolite levels on NPH

**Figure 3.**
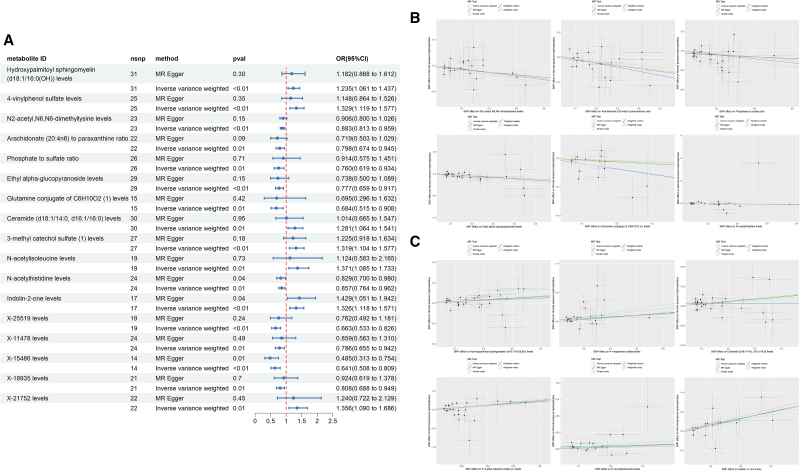
The association between plasma metabolite levels and NPH using 5 MR methods. (A) Forest plot of causality between 17 plasma metabolite levels and NPH. (B) Scatter plots of 6 protective factors in the known plasma metabolite levels. (C) Scatter plots of 6 risk factors in the known plasma metabolite levels. MR = Mendelian randomization, NPH = normal pressure hydrocephalus.

We conducted a 2-sample MR analysis using plasma metabolite levels as the exposure and NPH as the outcome, with IVW as the primary method. After removing SNPs in linkage disequilibrium and excluding weak IVs, we retained 34,856 SNPs associated with 1400 plasma metabolites, and the lowest *F*-value was 19.503. The IVW method initially identified 17 plasma metabolite levels with causal effects on NPH, including 12 known plasma metabolite and 5 unknown ones. We then performed heterogeneity and pleiotropy assessments (Table 3). Among the known plasma metabolite levels, 6 were identified as risk factors for NPH, including but not limited to N-acetylisoleucine levels (OR = 1.371, 95% CI = 1.085–1.733, *P*_adj = .01), and Indolin-2-one levels (OR = 1.326, 95% CI = 1.118–1.571, *P*_adj = .005). There were also 6 protective factors for NPH, including but not limited to glutamine conjugate of C6H10O2 (1) levels (OR = 0.684, 95% CI = 0.515–0.908, *P*_adj = .01), and phosphate to sulfate ratio (OR = 0.760, 95% CI = 0.619–0.934, *P*_adj = .01). Results from 4 additional MR methods supported the IVW findings for all 17 metabolites and revealed no signs of heterogeneity or horizontal pleiotropy (Fig. 3). Leave-one-out analysis confirmed that there were no outlier SNPs among the 12 known plasma metabolites (see Figure S2, Supplemental Digital Content 2, which demonstrates the result of the leave-one-out analysis).

**Table 3:** MR analysis of the causal relationship between plasma metabolite levels and NPH.

Exposure	Method	*N*snp	Beta	SE	*P*-val	*P*-val_adj	Pleiotropy	Heterogeneity
Hydroxypalmitoyl sphingomyelin (d18:1/16:0(OH)) levels	IVW	31	0.211	0.077	.006	.01	.75	.81
4-Vinylphenol sulfate levels	IVW	25	0.284	0.088	.001	.005	.22	.84
N2-acetyl, N6, N6-dimethyllysine levels	IVW	23	−0.124	0.042	.003	.008	.60	.98
Arachidonate (20:4n6) to paraxanthine ratio	IVW	22	−0.226	0.086	.009	.01	.53	.54
Phosphate to sulfate ratio	IVW	26	−0.274	0.105	.009	.01	.39	.93
Ethyl alpha-glucopyranoside levels	IVW	29	−0.252	0.084	.003	.008	.78	.95
Glutamine conjugate of C_6_H_10_O_2_ (1) levels	IVW	15	−0.380	0.145	.009	.01	.97	.30
Ceramide (d18:1/14:0, d16:1/16:0) levels	IVW	30	0.247	0.094	.009	.01	.24	.11
3-methyl catechol sulfate (1) levels	IVW	27	0.277	0.091	.002	.008	.52	.30
N-acetylisoleucine levels	IVW	19	0.316	0.119	.008	.01	.53	.21
N-acetylhistidine levels	IVW	24	−0.154	0.059	.009	.01	.59	.41
Indolin-2-one levels	IVW	17	0.282	0.087	.001	.005	.57	.77
X-25519 levels	IVW	19	−0.411	0.112	<.001	.002	.48	.65
X-11478 levels	IVW	24	−0.241	0.093	.009	.01	.65	.49
X-15486 levels	IVW	14	−0.445	0.119	<.001	.002	.17	.91
X-18935 levels	IVW	21	−0.213	0.082	.01	.01	.48	.41
X-21752 levels	IVW	22	0.305	0.111	.006	.01	.73	.24

IVW = inverse variance weighted, MR = Mendelian randomization, NPH = normal pressure hydrocephalus.

### 3.4. Mediation MR analysis among immune cell phenotypes, plasma metabolite levels and NPH

**Figure 4.**
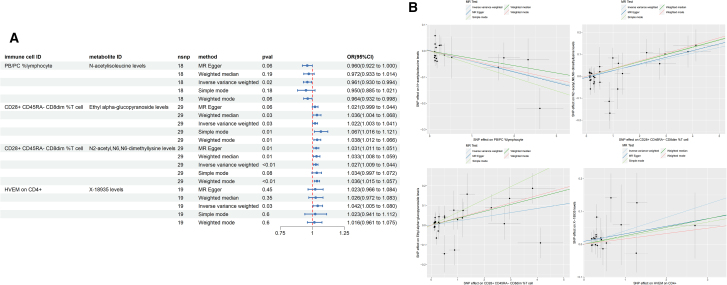
The association between 3 immune cell phenotypes and 4 plasma metabolite levels using 5 MR methods. (A) Forest plot of causality between immune cell phenotypes and plasma metabolite levels. (B) The causal effects of immune cell phenotypes on plasma metabolite levels in scatter plots. MR = Mendelian randomization.

**Figure 5.**
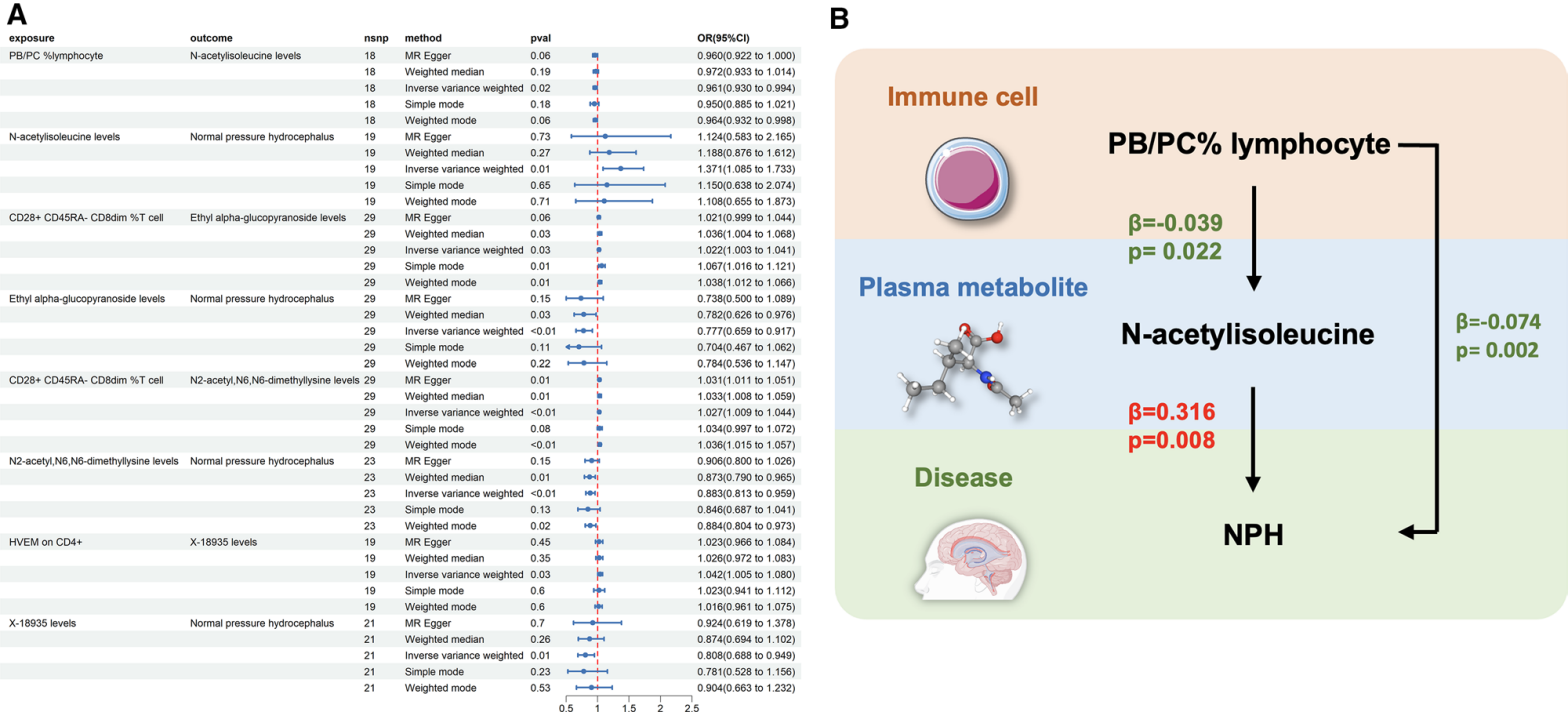
Mediation MR analysis among immune cell phenotypes, plasma metabolite levels and NPH. (A) Forest plot showing the causal relationships among the 3 immune cell phenotypes, the 4 plasma metabolite levels, and NPH. (B) N-acetylisoleucine levels mediate associations between PB/PC% lymphocytes and NPH. MR = Mendelian randomization, NPH = normal pressure hydrocephalus, PBs = plasmablasts, PC = plasma cells.

After establishing a causal relationship between immune cell phenotypes and plasma metabolite levels in relation to NPH, we conducted a mediation MR analysis to explore whether plasma metabolite levels mediate the relationship between immune cell phenotypes and NPH. To achieve this, we performed a 2-sample MR analysis, using the 5 identified immune cell phenotypes as exposure factors and 17 plasma metabolite levels as outcomes. This analysis uncovered a causal link between 3 immune cell phenotypes and 4 plasma metabolites, leading to an effect size Beta 1 from immune cell phenotypes to plasma metabolite levels, as detailed in Table 4. The leave-one-out analysis showed that no outlier SNPs influenced the overall results (see Figure S3, Supplemental Digital Content 3, which demonstrates the result of the leave-one-out analysis). Specifically, PB/PC% lymphocyte showed a protective effect on N-acetylisoleucine levels (OR = 0.961, 95% CI = 0.930–0.994, *P*_adj = .026), while CD28 + CD45RA-CD8dim% T cell was a risk factor for Ethyl alpha-glucopyranoside levels (OR = 1.022, 95% CI = 1.003–1.041, *P*_adj = .026) (Fig. 4). Next, we performed a MR analysis to assess the effect size Beta 2 from each of the 4 plasma metabolite levels to NPH. Figure 5A presents the causal relationships among the 3 immune cell phenotypes, the 4 plasma metabolite levels, and NPH. Based on the results, we calculated the overall effect Beta of each immune cell phenotype on NPH.

**Table 4:** MR analysis of the causal relationship between immune cell phenotypes and plasma metabolite levels.

Exposure	Outcome	Method	*N*snp	Beta	SE	*P*-val	*P*-val_adj	Pleiotropy	Heterogeneity
PB/PC %lymphocyte	N-acetylisoleucine levels	IVW	18	−0.039	0.017	.022	.026	.88	.12
CD28 + CD45RA-CD8dim %T cell	Ethyl alpha-glucopyranoside levels	IVW	29	0.021	0.010	.026	.026	.97	.39
CD28 + CD45RA- CD8dim %T cell	N2-acetyl, N6, N6-dimethyllysine levels	IVW	29	0.026	0.009	.003	.012	.37	.49
HVEM on CD4+	X-18935 levels	IVW	19	0.041	0.018	.026	.026	.45	.88

IVW = inverse variance weighted, MR = Mendelian randomization, NPH = normal pressure hydrocephalus.

### 3.5. Genetically predicted N-acetylisoleucine levels mediate associations between PB/PC% lymphocytes and NPH

In our analysis of the 4 potential mediators, we discovered that ethyl alpha-glucopyranoside, N2-acetyl, N6, and N6-dimethyllysine, and X-18935 showed effect directions inconsistent with the overall causal pathway. Consequently, they were not able to mediate the relationship between immune cell phenotypes and NPH. After integrating all results from the mediation MR analyses, we ultimately identified 1 valid mediator (Fig. 5B). PB/PC% lymphocyte showed a protective effect on N-acetylisoleucine levels (β = −0.039, *P* = .022, *P*_adj = .026), and higher N-acetylisoleucine levels increased the risk of NPH (β = 0.316, *P* = .008, *P*_adj = .01). PB/PC% lymphocyte also directly reduced the risk of NPH (β = −0.074, *P* = .002, *P*_adj = .006). The mediating effect was −0.0124, accounting for 16.8% of the total effect, and the direct effect was −0.062. These findings suggest that PB/PC% lymphocyte lowers the risk of NPH partly by reducing N-acetylisoleucine levels.

## 4. Discussion

In this study, we used a MR design to systematically explore the causal relationship between 731 immune cell phenotypes and NPH. Genetically predicted CD86 + myeloid DC AC, CD28 + CD45RA-CD8dim% T cell, and HVEM on CD4 + significantly increased the risk of NPH. PB/PC% lymphocyte, CD25++CD8br% CD8br, CD45RA-CD28-CD8br% T cell all protected against NPH. However, CD45RA-CD28-CD8br% T cell protection was weak, so it was not included in further analyses. NPH was not found to exert any effect on these immune cells. Previous studies have shown plasma metabolite levels as an important mediating factor between immune cells and allergic diseases.^[26]^ To explore the mechanism of different immune cells, we used MR to analyze 1400 plasma metabolite levels. We identified 17 plasma metabolite levels which are significantly associated with NPH. Among the 12 known plasma metabolites, 6 increased the risk of NPH, such as N-acetylisoleucine and indolin-2-one, while 6 were protective, such as ethyl alpha-glucopyranoside and phosphate to sulfate ratio. Interestingly, we found that N-acetylisoleucine is a mediating factor between PB/PC% lymphocyte and NPH, which means PB/PC% lymphocyte increased the protection effect of NPH by decreasing N-acetylisoleucine level (mediated ratio of 16.8%).

Previous studies have further shown that NPH is a neurological complication of several immune diseases, such as systemic lupus erythematosus,^[27]^ rheumatoid arthritis,^[28]^ myasthenia gravis,^[29]^ etc. These studies link NPH with the immune response, which is important for understanding the pathogenesis of NPH. Our study confirms a causal relationship between immune cell phenotypes and NPH, especially lymphocytes such as B cells and regulatory T cells (Tregs). Patients with idiopathic Normal Pressure Hydrocephalus often present with higher neutrophil counts and lower lymphocyte counts,^[30]^ reflecting an inflammatory state similar to that seen in other neurodegenerative diseases, such as Alzheimer disease and Parkinson disease.^[31]^

In our analysis, higher PB/PC% lymphocyte levels significantly reduced the risk of NPH. PBs and PCs are key components of the immune response. In normal conditions, the ratio of PB/PC of lymphocytes in peripheral blood is kept relatively high and supports effective antibody production.^[32]^ However, excess immunoglobulin production may lead to autoimmune response. PB/PC% lymphocytes alterations have been reported in several autoimmune diseases and cancers, among others. Both increases and decreases in PB/PC are associated with disease severity and prognosis.^[33]^ Some variations of PB/PC% lymphocytes can target neurons continually, which leads to neuroautoimmune diseases such as multiple sclerosis.^[34]^ PB/PC% lymphocytes also play an important role in immune regulation. PCs originate from activated B cells and are central to antibody production. Changes in the PB/PC may reflect the body’s response to immune stimulation such as infections and vaccinations. For example, PB/PC was found to increase after vaccination, which suggests a key role in antibody production and immune memory maintenance.^[35]^

The causes of hydrocephalus are diverse, including congenital, infectious, neoplastic and traumatic, in each of which the immune response is significantly different. Changes in PB/PC% lymphocytes are strongly associated with the etiology of hydrocephalus. In patients with infectious hydrocephalus, PB/PC% lymphocytes are markedly elevated,^[36]^ whereas, in tumor-associated hydrocephalus, immune suppression within the tumor microenvironment can alter lymphocyte profiles.^[37]^ Therefore, the analysis of PB/PC% lymphocytes may provide important clues for the etiological diagnosis of hydrocephalus. In addition, changes in PB/PC% lymphocytes not only reflect the immune status of patients with hydrocephalus, but also closely related to the prognosis of patients. Studies have shown that higher PB/PC% lymphocyte levels may be associated with severe disease and poor prognosis in patients with hydrocephalus.^[36]^ In some cases, high PB/PC% lymphocyte may affect the recovery of neurological function and quality of life by persistent inflammatory response.^[38]^ Although the role of PB/PC% lymphocyte in NPH is still unclear, this study shows that there is a causal relationship between them, and this finding may have greater significance for the intervention and treatment of NPH. As a potential biomarker of hydrocephalus, PB/PC% lymphocyte has important clinical application prospects. Future research should focus on the exploration of its specific mechanism and how it can be applied to the early diagnosis and treatment of hydrocephalus. This, in addition to providing patients with more precise medical solutions, will also promote the development of scientific research in related fields.

Additionally, we identified CD86 + myeloid dendritic cells (mDCs) as a risk factor for NPH. These cells play a critical role in the immune response, not only as key antigen-presentingcells but also in regulating T cell activation and differentiation, thereby influencing the strength and quality of immune responses.^[39]^ Beyond their immunological functions, mDCs also influence neurotoxicity, immune modulation, and tissue repair in the nervous system.^[34]^ In hydrocephalus, they may impact cerebrospinal fluid dynamics and ventricular enlargement by modulating the local immune environment and inflammatory response.^[40]^ Animal models of hydrocephalus have been used to explore the role of CD86 + mDCs, revealing significant changes in their number and function including upregulation of pro-inflammatory cytokines and increased immune cell infiltration. These are closely associated with the neurological damage and inflammatory response seen in hydrocephalus.^[41]^ Our findings are consistent with these observations and suggest that CD86 + mDCs play an active immunomodulatory role in NPH. These insights support the idea that immune dysregulation is present in NPH and that targeting CD86 + mDCs may offer new therapeutic opportunities. In recent years, immunotherapy has shown promising potential in the treatment of hydrocephalus. Targeting CD86 + mDCs to restore normal immune function and reduce inflammation in NPH patients could be a promising strategy. For instance, therapies targeting CD86 may promote neuroprotection and repair. Furthermore, combining CD86-targeted therapies with other immunotherapeutic approaches, such as cytokine therapy or monoclonal antibodies, could enhance treatment outcomes.^[42]^

Tregs represent another key component of immune regulation. They maintain immune tolerance, prevent autoimmunity, and suppress excessive inflammatory responses through the secretion of IL-10, TGF-β, and direct cell-to-cell interactions.^[43,44]^ Their role in the nervous system is equally significant. Tregs limit CNS inflammation, promote neuronal survival, and support neurological recovery.^[45,46]^ For instance, after acute intracerebral hemorrhage, Treg cells suppress neuroinflammation by secreting IL-10, reducing subsequent neuronal damage and dysfunction.^[47]^ In hydrocephalus, Treg cells may mitigate its progression and the associated neurological damage by modulating the local immune environment and suppressing excessive inflammation.^[48]^ Aquaporin 4 (AQP4), an aquaporin widely expressed in the nervous system, plays a role in glymphatic system function. AQP4 depolarization can lead to glymphatic dysfunction^[49]^ and contribute to the development of NPH.^[50]^ AQP4-deficient mice show significantly reduced Treg numbers,^[51]^ suggesting that AQP4 deficiency may impair Treg-mediated inflammatory inhibition and worsen NPH pathology. Our study found that CD25++CD8br% CD8br cells had a protective effect on NPH. However, CD28 + CD45RA-CD8dim% T cells and HVEM on CD4+ were associated with an increased risk of NPH. An imbalance in T cell subsets may further contribute to the progression of neuroinflammation.^[52]^ For example, the absence of Treg cells has been shown to exacerbate inflammation following chronic cerebral ischemia and worsen the pathological state.^[53]^ Moreover, in many neurodegenerative diseases, Treg cells lose their ability to suppress inflammation, instead contributing to a persistent pro-inflammatory environment.^[54]^ Exhaustion-like Treg phenotypes have been reported in systemic lupus erythematosus and ulcerative colitis, further diminishing their immunosuppressive capacity.^[55,56]^ The role of T cell subset imbalances in NPH requires further clinical investigation. Enhancing the function or number of Treg cells could offer a promising therapeutic approach for hydrocephalus and related neuropathological conditions.

Plasma metabolite levels can serve as predictors of disease outcomes.^[57]^ In our study, we found that the effect of PB/PC% lymphocytes on NPH was mediated by N-acetylisoleucine levels. PB/PC% lymphocytes were negatively correlated with N-acetylisoleucine levels, while N-acetylisoleucine levels were positively correlated with NPH. This suggests that reducing N-acetylisoleucine levels could enhance the protective effect of PB/PC% lymphocytes against NPH. N-acetylisoleucine is an N-acetylated derivative of isoleucine. High levels of this metabolite can damage nerves and the liver.^[58]^ Aminoacylase 1 is a soluble cytosolic homodimeric zinc-binding enzyme responsible for breaking down neutral N-acetylated amino acids.^[59]^ It plays a vital metabolic role in the nervous system. Aminoacylase 1 deficiency leads to the accumulation of N-acetyl amino acids in urine and leads to various neurological symptoms such as psychomotor delay, seizures, and intellectual disability.^[60,61]^ It can also affect immune function.^[62]^ Studies have also shown that N-acetyl amino acids disrupt mitochondrial bioenergetics and Ca^2+^ homeostasis in the brain,^[59]^ which may promote neurodegeneration. Therefore, the excessive accumulation of N-acetylisoleucine may contribute to NPH, and promoting its metabolism could be a potential therapeutic strategy. However, little is known about how PB/PC% lymphocytes influence N-acetylisoleucine levels. We speculate that N-acetylisoleucine may regulate amino acid metabolism and thereby modulate immune cell metabolic activity. These changes could support the protein synthesis required for B-cell differentiation into plasmablasts and plasma cells. This small peptide may also modulate macrophage activation and alter inflammatory cytokine levels, which would change the inflammatory environment and affect B-cell activation. Additionally, pathways related to amino acid transport and mitochondrial homeostasis may influence the differentiation process and ultimately shift the PB/PC% proportion.

In addition to N-acetylisoleucine, we identified other metabolites that showed significant effects on NPH, although they did not participate in the mediation process. For instance, elevated levels of Hydroxypalmitoyl sphingomyelin (d18:1/16:0(OH)) increased the risk of NPH. This metabolite is a derivative of sphingomyelin and a critical component of the myelin sheath. Myelin sheath plays an essential role in nerve impulse transmission, presynaptic plasticity, and neurotransmitter receptor localization.^[63]^ In NPH patients with excessive Aβ protein accumulation, inhibiting sphingomyelin synthase significantly reduced Aβ levels.^[64]^ Moreover, our study found that Ethyl alpha-glucopyranoside had a protective effect on NPH. Ethyl alpha-glucopyranoside is a derivative of alpha-glucopyranoside, which shows neuroprotective effects by reducing oxidative stress during ischemia.^[65]^ In addition, β-cyclodextrin, a compound made up of 7 α-D-glucopyranoside units, can reduce fibrosis and the neurotoxicity of Aβ.^[66]^ These observations suggest that Ethyl alpha-glucopyranoside may function as an intermediate that reduces Aβ toxicity, ultimately protecting against NPH. Unfortunately, there is limited research on the relationship between these metabolites and NPH, so further studies are needed to validate these findings.

Our study is the first to use mediation MR to investigate the causal relationship between immune cell phenotypes, plasma metabolite levels, and NPH. This provides novel insights into the pathogenesis of NPH and offers theoretical support for exploring potential therapeutic targets. However, clinical translation requires further validation of the stability and predictive value of these indicators in clinical research. In addition, if specific immune cell phenotypes or metabolic pathways represent actionable targets in NPH, they may offer new avenues for developing targeted therapeutic strategies. Our study has some limitations. First, all data were derived from the European population, limiting the generalizability of our conclusions. Research in diverse ethnic groups is needed. Second, the dataset lacked individual patient information, precluding further subgroup analysis. Finally, external validation in additional independent cohorts is still required to enhance the robustness of our conclusions. Although we identified a potential mechanism contributing to NPH, further basic and clinical research remains essential.

## 5. Conclusion

This study evaluated the relationship between immune cell phenotypes, plasma metabolite levels, and NPH. We identified a causal relationship between 6 immune cell phenotypes, 17 plasma metabolite levels and NPH. In addition, we used mediation MR analysis to reveal the protective effect of PB/PC% lymphocyte on NPH by decreasing N-acetylisoleucine levels, providing new insights into the mechanism of NPH development.

## Acknowledgments

We would like to thank the researchers who provided the GWAS dataset and acknowledge the participants and investigators of the FinnGen study. Special thanks to the researchers of the IEU GWAS database project for making the GWAS data publicly available. The public availability of the dataset enabled us to discuss the causal relationships of interest. Also, thanks to the National Center for Biotechnology Information, for providing the 3D structural image of N-acetylisoleucine (N-acetylisoleucine | C8H15NO3 | CID 306109 - PubChem (nih.gov)), which was utilized in this study.

## Author contributions

**Conceptualization:** Chang Liu.

**Data curation:** Chang Liu, Junqiang Wang, Cheng Wang.

**Formal analysis:** Junqiang Wang, Peng Long.

**Funding acquisition:** Gelei Xiao.

**Investigation:** Junqiang Wang, Yexin Yuan, Peng Long.

**Writing – original draft:** Chang Liu, Yexin Yuan.

**Writing – review & editing:** Gelei Xiao, Cheng Wang.

**Figure:**
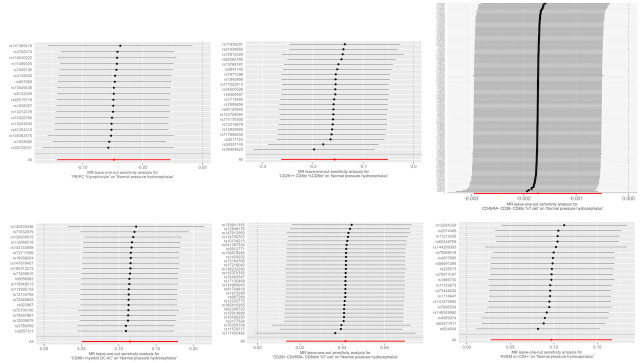


**Figure:**
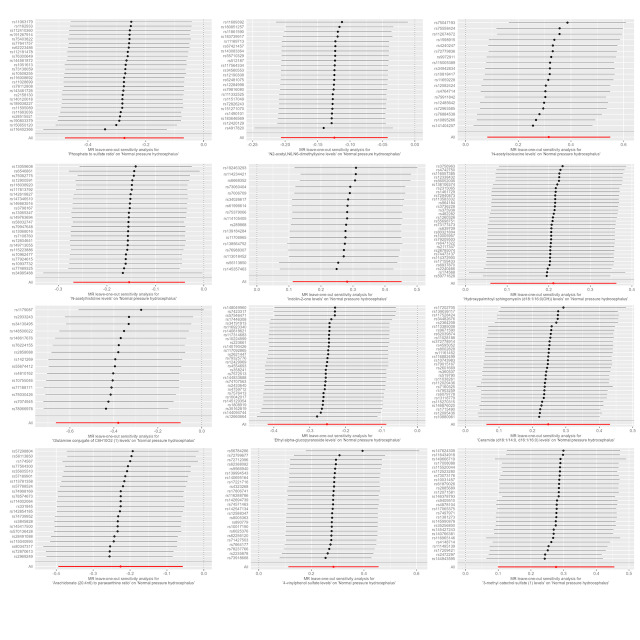


**Figure:**